# Methodologies to Evaluate the Micromechanics Flexural Strength Properties of Natural-Fiber-Reinforced Composites: The Case of Abaca-Fiber-Reinforced Bio Polyethylene Composites

**DOI:** 10.3390/polym15143137

**Published:** 2023-07-24

**Authors:** Faust Seculi, Fernando Julián, Joan Llorens, Francisco X. Espinach, Pere Mutjé, Quim Tarrés

**Affiliations:** 1 LEPAMAP-PRODIS Research Group, University of Girona, 17003 Girona, Spain; fernando.julian@udg.edu (F.J.); francisco.espinach@udg.edu (F.X.E.); pere.mutje@udg.edu (P.M.); 2 CATS Research Group, Department of Architecture and Construction Engineering, University of Girona, Avda Mª Aurelia Capmany 61, 17071 Girona, Spain; joan.llorens@udg.edu

**Keywords:** flexural strength, bio-based polymers, natural fibers, intrinsic properties

## Abstract

There is growing emphasis on developing green composites as a substitute for oil-based materials. In the pursuit of studying and enhancing the mechanical properties of these composites, tensile tests are predominantly employed, often overlooking the flexural properties. This study focuses on researching the flexural properties of abaca-fiber-reinforced bio-based high-density polyethylene (BioPE) composites. Specifically, composites containing 30 wt% of abaca fiber (AF) were treated with a coupling agent based on polyethylene functionalized with maleic acid (MAPE). The test results indicate that incorporating 8 wt% of the coupling agent significantly improved the flexural strength of the composites. Thereafter, composites with AF content ranging from 20 to 50 wt% were produced and subjected to flexural testing. It was observed that flexural strength was positively correlated with AF content. A micromechanics analysis was conducted to evaluate the contributions of the phases. This analysis involved assessing the mechanical properties of both the reinforcement and matrix to facilitate the modeling of flexural strength. The findings of this study demonstrate the feasibility of replacing oil-based matrices, such as high-density polyethylene (HDPE), with fully bio-based composites that exhibit comparable flexural properties to their oil-based counterparts.

## 1. Introduction

Musa textilis, commonly known as abaca, is a natural fiber that has wide applications in the paper industry, teabags, filters [1], ropes, twines, and nets [2]. Moreover, due to its high mechanical strength [3] and water resistance, it is a valuable component in the textile industry [4]. Being of natural origin, it is environmentally friendly in terms of sourcing and disposal, and its lightweight nature can contribute to cost savings [5]. These properties position abaca as an excellent candidate for the development of eco-friendly composite materials that combine natural reinforcements with bio-based plastics as matrices [6,7,8].

Previous studies have demonstrated the successful use of abaca as reinforcement in composites, particularly when combined with polypropylene [1]. Comparisons with glass fiber composites have revealed similar values for flexural strength and modulus, highlighting some advantages of abaca over glass fiber, such as ease of recycling [9], improved durability, and operator safety [10,11]. Abaca fiber composites are already in use as applications in the automotive industry [12,13] and are studied as potential building materials [14,15].

Earlier investigations reviewed the mechanical performance of thermoplastic reinforced with abaca fibers, with significant improvements in flexural strength observed in composites treated with maleic anhydride polypropylene copolymer (MAPP) [16,17,18]. The use of coupling agents in natural-fiber-reinforced composites has been extensively studied, demonstrating their ability to enhance tensile strength and Young’s modulus [19]. Among various chemical treatments and coupling agents, MAPE has been shown to enhance the flexural properties of high-density polyethylene (HDPE)-based composites [20].

Bio-polyethylene (BioPE) is a non-biodegradable bio-based plastic widely utilized on the market [21]. BioPE is obtained from ethylene generated through the fermentation and distillation of sugarcane juice, which produces ethanol [22], and is also produced from lignocellulosic biomass [23]. Its mechanical and chemical properties are similar to those of HDPE [24], which is renowned for its excellent mechanical and chemical properties [25]. Since both materials have similar properties, the use of BioPE is based on reducing the environmental impact of the composite obtention.

While prior work showed how biocomposites from abaca and polypropylene had reached the flexural strength and modulus of fiberglass-reinforced composites [1] to replace them, the substitution of the oil-based matrix (PP) with a bio-based one (BioPE) further reduces the environmental impact of the materials.

Previous research has focused on the use of Musa Textilis (abaca) as reinforcement in HDPE and BioPE composite materials, evaluating the tensile strength, tensile stiffness, and micromechanical characteristics of the composites [26,27]. These studies confirmed that, as expected, these materials exhibit superior mechanical properties compared to the matrix [28,29]. Moreover, adding natural reinforcement reduces the cost of the composite due to the low cost of the reinforcement compared to the matrix while enhancing its properties. Consequently, abaca and BioPE composites can be deemed promising alternatives to commercial oil-based materials.

To achieve comprehensive characterization, it is crucial to study the flexural properties of these composites. Understanding flexural behavior is highly significant, as bending forces are commonly encountered in practical applications, especially in structural applications [30]. While there are publications on the flexural properties of natural fiber-reinforced polymer composites [30,31,32], the number of publications analyzing the flexural properties of abaca fiber and plastic matrix composites remains limited [1,33]. This indicates the need for further exploration of the flexural characterization of such composites.

Natural fiber composites need treatments to improve the interface between the matrix and the reinforcement [34,35]. Different kinds of fiber transformation have been proven to provide strong bonding, such as chemical treatment [36], alkaline treatment [37], magnesium oxide and zeolite [38], enzyme modification [39], and MAPE [40].

As demonstrated in previous articles [41,42], the use of coupling agents facilitates the establishment of a strong interface between the hydrophilic reinforcement and the hydrophobic matrix [33,43]. Maleic-acid-based coupling agents are commonly employed for this purpose [44]. In this study, flexural tests were conducted on composites containing 30 wt% of abaca fiber, with varying amounts (0% to 10%) of MAPE.

**Figure 1:**
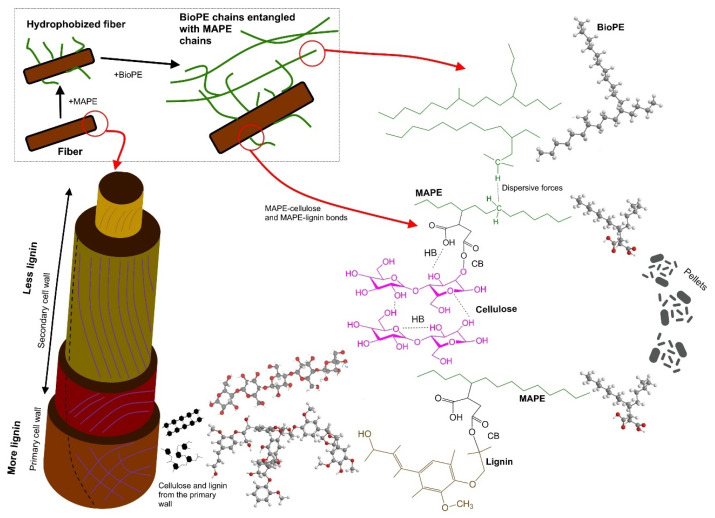
Diagram of the fiber, BioPE, and MAPE interaction.

Even though abaca fibers are overall rich in cellulose and have a relatively low lignin content (roughly from 5 to 13 wt%) [45], the latter is more abundant in the outer layers of the fibers, namely the primary cell wall and the first layer of the secondary cell wall. In the absence of chemical treatments, the outer layers remain mostly intact, and thus most surface interactions involve the primary cell wall. Therefore, covalent bonding (CB in Figure 1) between MAPE and lignin may be as important as that between MAPE and cellulose. MAPE-lignin CB may take place either through the esterification of exposed hydroxyl (-OH) groups, transesterification of methoxy (-OCH_3_) groups, or both. In any case, as estimated in a previous work of ours for MAPP and polypropylene [46], the simultaneous bonding of the two reactive oxygen atoms of each anhydride moiety is thermodynamically hindered. Instead, the unreacted end becomes able to establish hydrogen bonds (HBs), which, due to the lower density of hydroxyl groups in lignin, are expected to be more relevant in the case of cellulose (Figure 1).

MAPE-modified fibers can then be dispersed in a BioPE matrix through physical entanglement, in the same way polyethylene chains are self-entangled in pure BioPE. Although certain kinds of intermolecular forces exist between chains, these are weak dispersive interactions. The reasons for chemical and/or electrostatic nature are outweighed by simple polymer physics: chains crossing the same plane multiple times restrain the movement of the rest of the chains. Fiber aggregation is then avoided by the fact that the polyethylene chains attached to them (MAPE-fiber), the polyethylene chains of another MAPE-modified fiber in proximity, and the polyethylene chains in between (BioPE) are mutually entangled. The strength of the reinforcement directly impacts the strength of the composite material [47]. Micromechanics models, such as the rule of mixtures, can be employed to determine the intrinsic strength of the reinforcement or model the strength of a composite [19,48]. These models assist in quantifying the contribution of the fibers to the composite’s strength.

The objective of this study is to characterize the flexural behavior of abaca and BioPE composites. BioPE composite materials reinforced with 20–50% abaca content were produced to achieve this. These composites were then shaped into standard samples through injection molding and subjected to flexural stress testing. Furthermore, to assess the efficiency of the reinforcement and the contribution of different phases, a micromechanical analysis employing the rule of mixture and Hirsch’s equation was conducted.

The findings of this research highlight how adding abaca fibers is a promising approach for developing BioPE-based composites that possess desirable performance properties, cost competitiveness, and a reduced environmental footprint.

## 2. Materials and Methods

### 2.1. Materials

The matrix used to create the composite is a bio-based high-density polyethylene (BioPE) with reference SHA7260, a density of 0.955 g/cm^3^, and a melt flow index of 26 g/10 min (190 °C; 2.16 kg). It was acquired from Braskem (Sao Paulo, Brazil). The other matrix used to test is an HDPE with reference HDI0661U1 with a density of 0.953 g/cm^3^ and a melt flow index of 26 g/10 min (190 °C; 2.16 kg). The coupling agent used to improve the strength of the interface between the reinforcement and the matrix is polyethylene functionalized with maleic acid (MAPE). This material has a density of 0.96 g/cm^3^ and a melt flow index of 2.0 g/10 min (190 °C; 2.16 kg) [26]. The coupling agent was provided by DuPont (Wilmington, DE, USA), with the commercial reference Fusabond^®^ MB100D (Dow, Midland, MI, USA)

**Figure 2:**
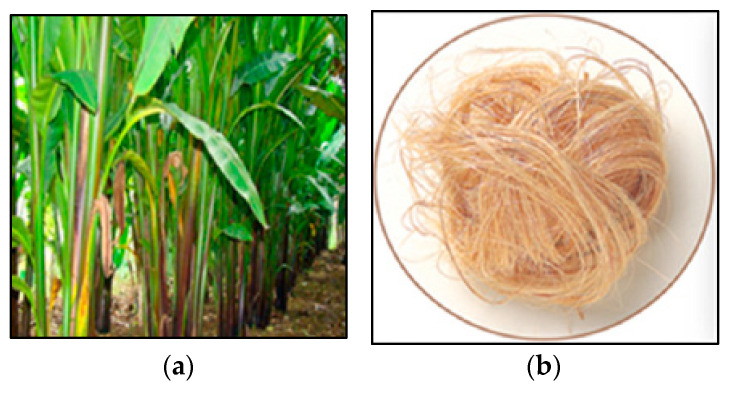
(**a**) Abaca plant; (**b**) fibers extracted by mechanical means.

The natural fiber used as reinforcement is a Filipino abaca strand (*Musa textilis*), supplied by CELESA (Tortosa, Spain) (Figure 2). The composition of these fibers was analyzed in a previous publication [26]. Abaca fibers were composed of 72.7 wt% cellulose, 14.6 wt% hemicellulose, 8.9 wt% lignin, 2.9 wt% extractives, and 0.9 wt% ashes.

### 2.2. Composite Fabrication

There was no previous chemical treatment applied to the abaca fibers. The reinforcement used to produce the composite was untreated virgin abaca bundles. To compound the phases, we used an intensive melt mixer Brabender^®^ Plastograph (Kulturstr, Germany). The mixing was carried out at 180 °C and 80 rpm for 10 min. These process parameters were used in previous articles to ensure good fiber dispersion and the proper mixing of the phases, and to avoid the attrition phenomenon of the fiber due to reinforcement shortening. After cooling down, the produced composites were introduced into a hammer mill to transform them into pellets. The mean diameter achieved after the process was 5 mm.

The pellets were stored at 80 °C for 24 h to remove the humidity before mold injection. The procedure used for obtaining flexural test specimens was injection molding [49], and dog bone standard specimens according to ASTM D638 were produced using a mold injection machine Meteor^®^ 40 (San Francisco, CA, USA) by Mateu and Solé (Barcelona, Spain).

In the tables, the materials are described as BioPE + X% Abaca + Y% MAPE, where X is the wt% of abaca fibers used as reinforcement of the composite and Y is the wt% (concerning the reinforcement) of MAPE.

### 2.3. Flexural Testing of the Composites

The flexural strength was determined through a three-point bending flexural test according to the standard ASTM D790 [50]. The samples used for the test were prepared according to the ASTM D760 standard [51]. At least 5 samples were used for all of the composite formulations. The samples were placed in an Instron^®^ 1122 Universal testing machine supplied by Metrotec, S.A. (Barcelona, Spain) fitted with a 5 kN load cell to determine the flexural strength and flexural deformation.

**Figure 3:**
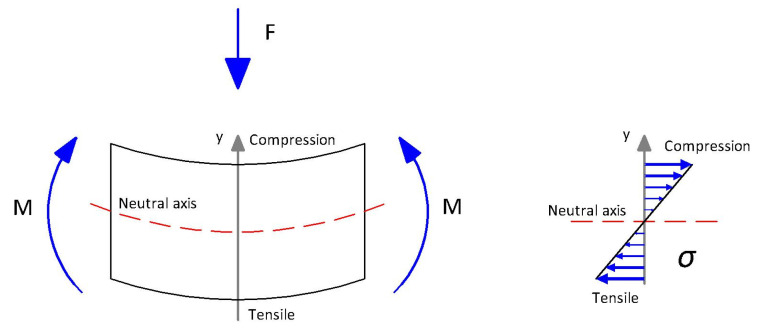
Stress distribution in a flexural test.

The composite material being subjected to flexural stress causes some of the fibers to work under tensile stress and some under compression stress, as shown in Figure 3. The force (F) is applied to the sample, causing the formation of two flexural moments (M) on both edges. The sum of both moments counteracts the force. The force acts on the axis Y and is perpendicular to the neutral axis. The neutral axis passes through the center of gravity of axis Y. Axis Y determines what part of the sample is working under compression and what part is under tensile stress. This phenomenon leads to a higher value of flexural strength than tensile strength because the fibers are all working under compression or tensile stress.

To determine the highest flexural strength that MAPE content can provide, two-stage tests have been developed. Uncoupled composites with 30 wt% of abaca fibers and BioPE matrix were prepared and tested. Hereafter, composites containing the coupling agent MAPE with a content ranging from 2 wt% to 10 wt% were prepared and tested. The percentage of MAPE was calculated against AF content. In addition, a virgin BioPE sample was prepared and tensile-tested to compare it with prepared and tensile tested composites containing abaca fibers from 20 to 50 wt% and a permanent MAPE content of 8 wt% for all the samples.

All the experimental results were analyzed using ANOVA [31] with R^®^ and RCommander with a confidence interval of 95%.

**Figure 4:**
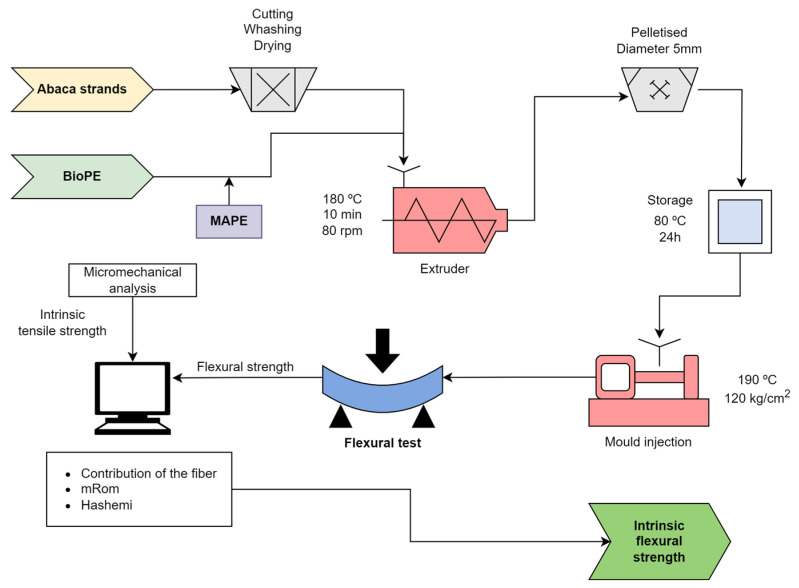
Diagram of the methodology, including mixing, injection, and flexural test (to be combined with the outputs from the tensile tests) [26,27,52].

Figure 4 shows a flowchart of the research.

## 3. Flexural Strength Micromechanics Models

The use of a rule of mixtures (mRom) for an aligned short-fiber-reinforced polymer composite is well explored in previous publications [53]. It was originally developed for Young’s modulus, but it can be adapted in the case of flexural strength [54] as follows:

(1) $$\sigma_{f}^{C}=\sigma_{f}^{F}\cdot V^{F}+\left(1-V^{F}\right)\cdot \sigma_{f}^{M}$$

where the flexural strengths of the composite, reinforcement, and matrix are represented by $\sigma_{f}^{C}$, $\sigma_{f}^{F}$, and $\sigma_{f}^{M}$, respectively, and $V^{F}$ is the volume fraction of the reinforcement.

A modified version of the rules of mixture can account for the strength of the interface, the morphology of the reinforcements, and their mean orientation with the loads as follows:

(2) $$\sigma_{f}^{C}=f_{f}^{F}\cdot \sigma_{f}^{F}\cdot V^{F}+\left(1-V^{F}\right)\cdot \sigma_{f}^{m*}$$

where $\sigma_{f}^{m*}$ is the contribution of the matrix at the ultimate flexural of the composite and $f_{f}^{F}$ is a coupling factor. This efficiency factor includes the effects of the strength of the interface between the matrix and the reinforcement, mean fiber orientation, and fiber length. The values $V^{F}$ and $\sigma_{f}^{m*}$ are determined by experimental methods, and the remaining values $f_{f}^{F}$ and $\sigma_{f}^{F}$ are two unknowns. These two mechanical properties are difficult to measure experimentally due to the difficulty of evaluating the mechanical and morphological properties of fibers individually. The results display high dispersion. The preferred methodology by some authors is micromechanical methods [55]. The coupling factor accounts for the strength of the interface, the fiber’s mean orientation, and its morphology. Its value varies from 0.18 to 0.2, according to the literature [56].

Equation (2) has two unknown values, the intrinsic flexural strength of the fiber and the coupling factor. Both unknowns are interrelated. Due to the difficulty of measuring these two unknowns on an experimental basis, we used micromechanics methods.

Equation (2) can be reworked as shown in Equation (3) [57]:

(3) $$FFSF=\frac{\sigma_{f}^{c}-\left(1-V^{F}\right)\cdot \sigma_{f}^{m*}}{V^{F}}=\bar{f_{c}\cdot \sigma_{f}^{F}}$$

where *FFSF* is a fiber flexural strength factor and it is the slope of the regression line of the contribution of the fibers at different contents, representing $\sigma_{f}^{c}-\left(1-V^{F}\right)\cdot \sigma_{f}^{m*}$ against $V^{F}$ at each fiber content. This is the measure of the potential strengthening capabilities of any reinforcement, as has been used in previous articles [58]. To find a theoretical value of the intrinsic tensile strength, we used the Hirsch equation [53]:

(4) $$\sigma_{f}^{C}=\beta \cdot \left(\sigma_{f}^{F}\cdot V^{F}+\sigma_{f}^{m*}\cdot \left(1-V^{F}\right)\right)+\left(1-\beta \right)\cdot \frac{\sigma_{f}^{F}\cdot \sigma_{f}^{m*}}{\sigma_{f}^{m}\cdot V^{F}+\sigma_{f}^{F}\cdot \left(1-V^{F}\right)}$$

where β is an efficiency factor that accounts for the mean orientation of the fibers and the load transfer between the fibers and the matrix. In publications where the Young’s modulus of composites reinforced with semi-aligned short fibers is analyzed, a value of β = 0.4 is proposed as the most appropriate to experimental values [53,59]. In the case of flexural strength, an efficiency factor of 0.15 for composite materials with semi-aligned short fibers is the value that fits better, as we will show later on in this paper. We determined the value through iteration based on spherical particles having a value of 0.1 [60].

The objective of this study is to determine the value of the flexural strength of the composite ($\sigma_{f}^{C}$) and the strain at the maximum flexural strength ($\varepsilon_{f}^{c}$). Both values are determined in two different cases. The first concerns the MAPE content of the composite. The second is once an optimal percentage of MAPE is designated against the content of abaca fibers. The way to achieve this objective is through experimental methods and modeling. 

The value of deformation (D) is determined during the three-point bending test. The maximum flexural strain ($\varepsilon_{f}^{c}$) was obtained using Equation (5):

(5) $$\varepsilon_{f}^{c}=\frac{6\cdot D\cdot d}{L^{2}}$$

where d is the specimen width with a value of 3.1 mm and L is is the support span of the sample with a value of 52.6 mm.

The modified rule of mixtures has two unknowns that are not possible to obtain from the experimental test, $f_{f}^{F}$ and $\sigma_{f}^{F}$. Using micromechanics modeling, it is possible to find the value of $\sigma_{f}^{C}$. We have used three different models to obtain theoretical values for the unknowns. The three models are as follows:

The Hashemi correlation: equation defined by [33,61]:

(6) $$\sigma_{f}^{F}=\frac{\sigma_{f}^{C}}{\sigma_{t}^{C}}\cdot \sigma_{t}^{F}$$

where the flexural strength of the composite and the reinforcement are represented by $\sigma_{t}^{C}$ and $\sigma_{f}^{F}$, respectively, and the tensile strength of the composite and the reinforcement are represented by $\sigma_{t}^{C}$ and $\sigma_{f}^{F}$, respectively.

The contribution of the fibers: the modified rule of mixtures is applied in order to determine the contribution of the phases to the composite’s strength [33,46]:

(7) $$\sigma_{f}^{F}=\frac{FFST}{FTSF}\cdot \sigma_{t}^{F}$$

Hirsch’s equation: the value of $\sigma_{t}^{C}$ is set by using Equation (4). Defining factor β has significant importance. Its value is established for tensile strength cases, but not for flexural strength. β cannot be the same value in both kinds of loads, due to samples being subjected to compressive stresses and tensile stresses in flexural load. 

With these three models, we have obtained three different values of $\sigma_{f}^{F}$. Afterward, factor $f_{f}^{F}$ is determined for each model using Equation (2).

## 4. Results and Discussion

### 4.1. Characterization of the Reinforcing Fibers

**Figure 5:**
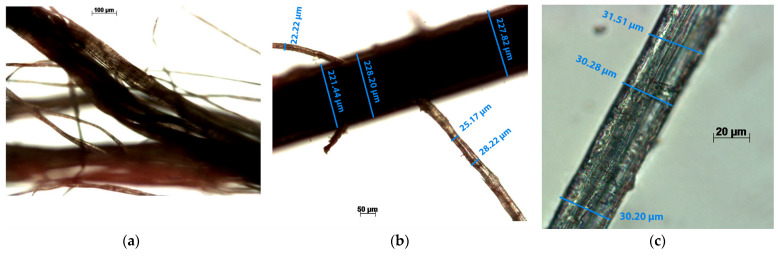
Micrographs of abaca fibers: (**a**) fiber bundle; (**b**) comparison between the diameter of a fiber bundle and an individualized fiber; (**c**) detail of a fiber.

To determine the diameter of the abaca strands, an optical microscope was utilized in this study. Subsequently, an optical image analysis was conducted, providing a comprehensive assessment of the string’s diameter. The inspection revealed a diameter range spanning from 25.17 µm to 31.51 µm, as illustrated in Figure 5.

The strands of abaca were examined using an optical microscope Zeiss AXIO model Scope.A1 (Oberkochen, Germany). The abaca strands were frayed to select a single string and analyze it. With this procedure, the width of a string was determined and its rough surface was observed.

Figure 5 shows how the abaca bundles are arranged (a and b), and the rough surface of an abaca strand (c) in microscopic images.

### 4.2. Effect of MAPE Coupling Agent on Flexural Properties

The inherent differences between the matrices and the natural fibers result in an incompatible interface, primarily due to the hydrophilic nature of the fiber and the hydrophobic nature of the matrix. To address this issue and improve the mechanical properties of the composite, a coupling agent is introduced into the mixture [62]. A previous study extensively discussed the effects of incorporating MAPE into abaca fiber with BioPE and HDPE composites, specifically focusing on the tensile properties.

From previous publications [26,27], which investigated the mechanical properties of composites with varying percentages of abaca, it was determined that a reinforcement content of 30 wt% AF yields favorable results for conducting general experimentation.

Based on this determination, the present study focused on measuring the flexural properties of the composites, including flexural strength, deformation, and elongation at break. The corresponding results are presented in Table 1.

$\sigma_{f}^{m*}$ can be obtained from the values of the stress–strain curve of the matrix, which yields the following equation: $\sigma_{f}^{m*}$ = −0.0107x^4^ + 0.2283x^3^ – 1.9883x^2^ + 9.4707x + 0.0052, where x is the corresponding $\varepsilon_{f}^{c}$.

Upon analyzing the measurements of deformation and elongation at break, we observed a strong correlation between these two parameters, with a coefficient close to 1.

The comparison between the flexural strength value of the BioPE ($\sigma_{f}^{C}$ = 21.25 MPa) and the value of the sample with no coupling agent ($\sigma_{f}^{C}$ = 30.11 MPa) gave a higher value than the last one. This means the adhesion of the reinforcement and the matrix works. According to our previous research, the coupling occurs using mechanical anchoring between the rough surface of the reinforcement [24] and the matrix. The surface of the fiber is like a hillside ridge [63,64]; thus, the matrix fills the valleys and surrounds the whole bundle when it is disseminated on the surface of the fiber. Another possibility explaining the union phenomena is the effect of the extractives inside the fiber [65], which are hydrophobic in nature as is the matrix, inducing both materials to bond.

The flexural strength increases with the addition of the coupling agent. The maximum strength achieved by the composite was 50.28 MPa, with a MAPE content of 8 wt%. This value is two and a half times higher than the non-reinforced matrix, approximately the same as the case of the HDPE matrix, with a value of $\sigma_{f}^{C}$ = 22.28 MPa.

The tensile strength of the composite with 30 wt% of AF and 8 wt% content had a value $\sigma_{f}^{C}$ = 33.08 MPa in previous work [27]. Comparing it with the value of the flexural strength for the same composite $\sigma_{f}^{C}$ = 50.28 MPa, the flexural strength is 1.52 times higher than the tensile strength, confirming the expectations explained before about samples under flexural stress.

**Figure 6:**
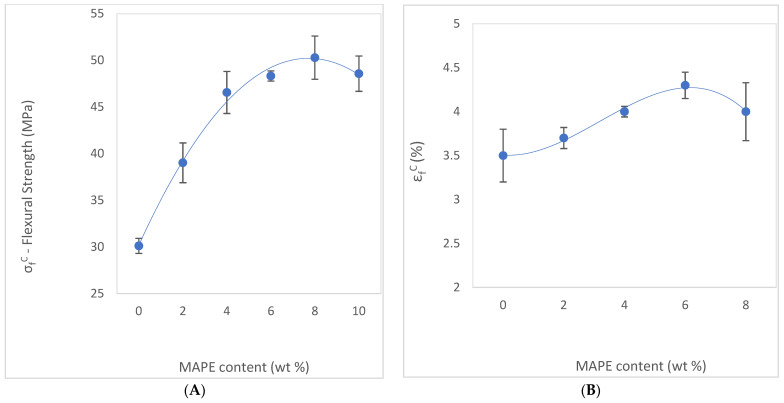
(**A**) Effect of MAPE content on the flexural strength of abaca-reinforced composites (the error bars indicate three times the standard deviation). (**B**) Effect of MAPE content on the elongation at break of abaca-reinforced composites (the error bars indicate three times the standard deviation).

Figure 6A shows the effectiveness of the MAPE coupling agent addition to the composite. It is shown how the increases in MAPE caused a relevant increase in the composite flexural strength. All MAPE grades result in improvement concerning the AF-reinforced BioPE. For instance, BioPE with 30% wt% AF without MAPE has a flexural strength of 30.11 MPa, whereas with MAPE, the flexural strength varies from 39.01 to 50.28 MPa.

It is noteworthy that up to 8 wt% MAPE, there is a consistent increase in flexural properties, arriving at its higher value of 50.28 MPa. From this value upwards, the properties decrease, meaning that the optimal MAPE content value is the 8 wt% marked as the top properties performance.

Even with similar values of flexural strength with MAPE content ranging from 4 wt% to 8 wt%, the value differences among them are relevant, as the ANOVA analysis determined. The decrease in flexural strength at a MAPE content of 10 wt% is explained by the self-entanglement occurrence of MAPE molecules caused by the saturation of the hydroxyl groups. The decrease in flexural strength is usually attributed to the migration of excess MAPE around the fiber surface, resulting in slippage [60,62].

Analyzing the contribution of the matrix to the strength of the composite ($\sigma_{f}^{C}$/$\sigma_{f}^{m*}$), regarding MAPE content, we observe an initial improvement in the strength up to a MAPE content of 4 wt%, which means the increase in the matrix strength contributes to the composite strength. From 4 wt% to 10 wt% MAPE content, the contribution of the matrix stabilizes, showing almost the same values of strength.

Reviewing the contribution of MAPE to the strength of the composite, we have compared the composite material strength with different contents of MAPE ranging from 2 wt% to 10 wt% against the strength composite with 0 wt% of MAPE content. We have observed that the value of the ratio $\sigma_{f}^{C}$/$\sigma_{f}^{C}$ (0% MAPE) is always above 1, meaning that the composite flexural strength is improved with the addition of MAPE. Furthermore, from 4 wt% to 10 wt% MAPE content, the contribution of MAPE stabilizes, showing almost the same values. Examining the values in Table 1, it can be seen how the deformation capacity of the composite (with values from 3.4% to 4.3%) has been reduced compared with virgin BioPE (6.6%). Every composite sample containing MAPE shows a worse deformation capacity than the BioPE alone. The reason for this behavior is that the reinforcement is a more fragile and rigid element than the matrix, and its contribution to the composite causes an increase in stiffness.

Figure 6B shows how MAPE content has a slightly significant effect on deformation once introduced to the composite. The evolution of the values of deformation and elongation at break are quite similar. Both properties show a little improvement with the increase in MAPE, achieving the maximum value of elongation (4.3%) at 8 wt% MAPE content.

Summarizing the effect of MAPE addition, the composite had lost some capacity for deformation before the break. However, it increased the flexural strength in comparison with the matrix alone of BioPE and HDPE. Once an optimized content of MAPE was established, other composite material formulations were analyzed.

### 4.3. Effect of Fiber Content on Flexural Properties

After establishing the content of MAPE in the abaca-reinforced BioPE-based composite, we proceeded to test the flexural properties with different percentages of abaca fibers. Setting a constant MAPE content of 8 wt%, BioPE composites were reinforced with different percentages of abaca strands, ranging from 20 wt% to 50 wt%, according to the methodology explained previously. We present the results of the bending test as a function of the fiber loading in Table 2. *D* is the experimental deflection of the studied samples during the bending tests, and $\varepsilon_{f}^{c}$ is the deformation at the maximum flexural strength value.

**Figure 7:**
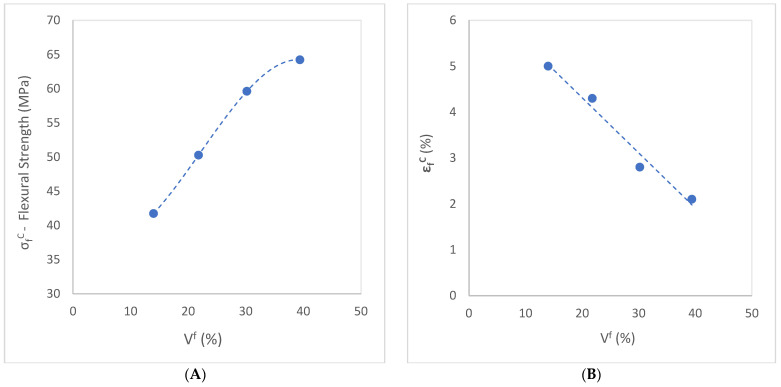
(**A**) Effect of reinforcement volume fraction on the flexural strength of abaca-reinforced composites. (**B**) Flexural stress–strain curve. Evaluation of the matrix contribution to the flexural strength of the composite.

The flexural strength is positively correlated, in a linear progression, to the abaca content. It reaches the maximum value of $\sigma_{f}^{C}$ = 64.23 MPa at 50 wt% content of abaca, which is up to three times higher than those of plain BioPE samples ($\sigma_{f}^{C}$ = 21.25 MPa) and HDPE samples ($\sigma_{f}^{C}$ = 22.28 MPa). This correlation indicates a homogeneous dispersion of the reinforcement in the matrix, meaning a good interface has been achieved. The results were expected according to similar values obtained by other authors [31,63]. Related to this, the deformation of the composite decreased, as did the elongation to break, as shown in Figure 7B.

Figure 7A shows how the flexural strength follows a quasi-linear evolution with the fiber volume fraction from 20 to 40 wt%. This evolution indicates satisfactory stress transfer between phases and proper dispersion of the reinforcement inside the matrix. Flexural strength achieves an inflection point at 40 wt% of AF content. While the slope is quite proportionally ascendant from 20 to 40 wt%, from that point on, the curve reduces its tendency to reach 50 wt% of AF content. From this percentage, adding more reinforcement content to the composite does not significantly improve the flexural properties.

Figure 7B shows the effect of the fiber volume fraction $V^{F}$ on the elongation at the break of the abaca-reinforced BioPE-based composite. The figure presents a reversed relationship between the deformation and the abaca content. Increasing the addition of rigid and fragile elements entails a decrease in the composite deformation properties.

**Figure 8:**
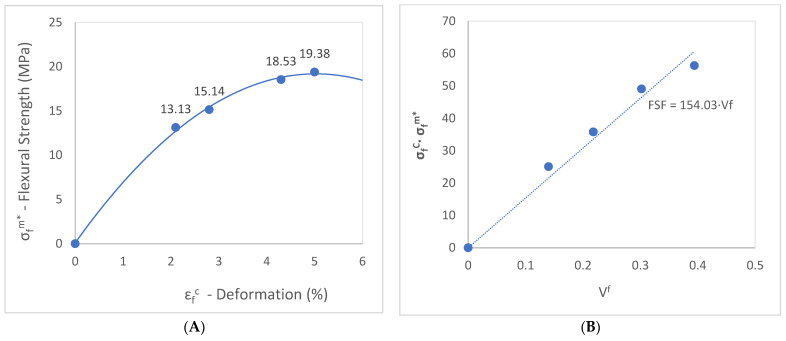
(**A**) Flexural stress–strain curve. Evaluation of the matrix contribution to the flexural strength of the composite. (**B**) Flexural strength factor of abaca-reinforced composite coupled with 8% MAPE.

Based on the values obtained in Table 2, Figure 8A displays the evaluation of the matrix contribution to the flexural strength of the composite.

The stresses supported by the matrix are obtained from the stress–strain curve. The difference between this value and the strength of the composite results in stress being transferred to the reinforcement. Consequently, it is possible to evaluate the intrinsic mechanical properties of the fibers inside the composite.

Figure 8B illustrates the fiber flexural strength factor (*FFSF*), represented by the slope of the curve obtained by the graphic $\sigma_{f}^{C}$/$\sigma_{f}^{m*}$ vs. $V^{F}$ (Equation (3)). This factor has been determined from the characteristics of the material and the characteristics of the injection molding system.

The result obtained for the fiber tensile strength factor (*FTSF*) for the coupled abaca-reinforced BioPE matrix in a previous publication was 100.89 [52]. The flexural strength factor (*FFSF*) is around 1.5 times higher than the tensile strength factor, with a value of 154.03, as was expected and published by previous authors [61]. This is because the composites exposed to flexural loads support a combination of compressive and tensile forces at the cross-sectional area of the samples during the bending test. Consequently, the fibers have a higher contribution to the flexural strength.

**Figure 9:**
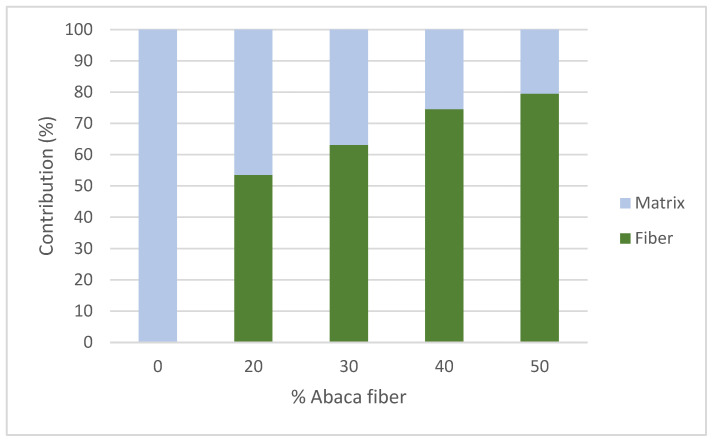
Contribution of the matrix and the reinforcement to the flexural strength of the composite at different content percentages.

Figure 9 shows the contribution to the flexural strength of the composite by the flexural strength of the reinforcement and the matrix and its evolution according to the fiber content. It is significant how the fiber provides more than 50% of the flexural strength from the lower value content, demonstrating the great importance of the reinforcement for the flexural strength of the composite material.

### 4.4. Modeling of the Flexural Strength of the Composites

#### 4.4.1. Modeling of the Composite regarding MAPE Content

The mRom was calculated through the three methodologies introduced in the modeling section, Equations (4), (6), and (7). Table 3 shows the obtained results.

Analyzing Table 3, we observed how two methods, Hashemi and fiber contribution, show a linear behavior, especially using the fiber contribution method, since it was developed using mean values. According to the contribution of the fiber approach, the result obtained is a single value of $\sigma_{f}^{F}$ = 785.1 MPa. The value of *FTSF* was obtained from a previous publication, being 100.89, and the value of *FFSF* was found before in this study, and it was 154.03. The value of $\sigma_{t}^{F}$ was also established in a previous publication, and its mean value was 514.25 [52].

The Hashemi method had a small variance, ranging from 689.4 MPa to 838.2 MPa, where we obtained 775 MPa as the mean value of $\sigma_{f}^{F}$.

Observing the values in Table 3 for the Hirsch approach, we found the value of the the intrinsic flexural fiber strength (IFFS) of the AF-reinforced BioPE is 312.8 MPa, whereas with MAPE, the IFFS varies from 573.3 to 858.9 MPa. The top value of the IFFS is found in the 30 wt% AF-reinforced BioPE with 8 wt% MAPE content. The same conclusion was found in the flexural strength of the composite concerning MAPE content. Therefore, from 8 wt% MAPE content, the phenomenon of self-entanglement of the MAPE occurs. Using the Hirsch approach, the mean value of $\sigma_{f}^{F}$ obtained is 696.1 ± 213.8 MPa. The optimal value of $\sigma_{f}^{F}$ is 858.9 MPa for the composite of BioPE with a 30% content of AF and 8 wt% of MAPE. The table shows that for MAPE percentages of 0 and 2% wt%, the intrinsic flexural strength is quantitatively lower than percentages ranging from 4 to 10 wt%, which have similar values. The reason for this phenomenon is explained by the method used to obtain the results, meaning this is a mathematical outcome and not a result of the physical and morphological nature of the composite. Considering the percentage of AF is the same, with a content of 30%, the values should be statistically similar.

**Figure 10:**
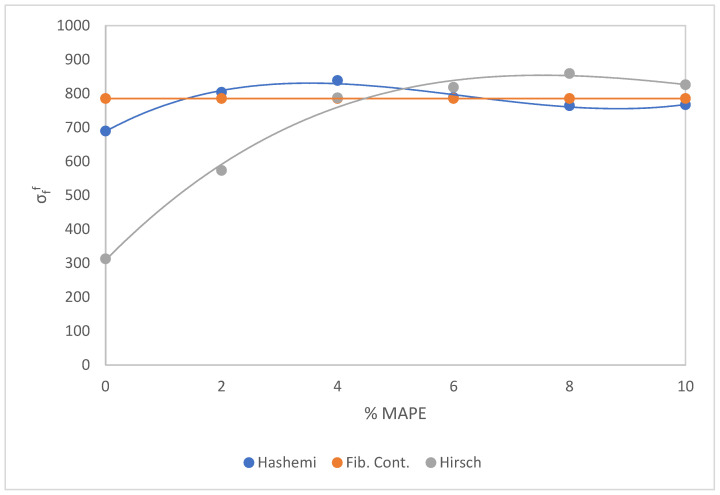
Effect of MAPE content on the intrinsic fiber strength, according to the three modeling methods.

Figure 10 shows the influence of the weight percentage of MAPE on the IFFS. All the MAPE grades result in improved flexural intrinsic fiber strength of the AF-reinforced BioPE.

Considering the evolution of the results shown in Table 3 and Figure 10, we concluded that a high percentage of MAPE, from 4 wt%, presents a higher exploitation of the AF stiffening capabilities. All three mean results obtained have similar values, 785.1 MPa, 775 MPa, and 696.1 ± 213.8 MPa, resulting in the confirmation that the three-model methodology used is correct and reliable. Using the mRom (Equation (2)), we determined the value of $f_{f}^{F}$ for all three methods. The mean value of $f_{f}^{F}$ is 0.18 according to the Hashemi approach, The mean value, of $f_{f}^{F}$ is 0.18 by the fiber contribution approach, and, in compliance with the Hirsch approach, the mean value of $f_{f}^{F}$ is 0.20. All the values range from 0.18 to 0.22, as expected.

Analyzing the values of $f_{f}^{F}$ obtained in Table 3 through the Hirsch equation, we have reached the same conclusion as for the intrinsic flexural strength. The $f_{f}^{F}$ values for MAPE percentages of 0 and 2% wt% are higher than expected. The reason is due to the use of a mathematical model. The values of $f_{f}^{F}$ shown in Table 3 for the Hirsch model lead to the fact that it is important to have profound knowledge of the model used and the possible outcomes to determine the reliability of the data obtained and to identify any disruptive value. Using the optimal value of $\sigma_{f}^{F}$ in the Hirsch Equation (4), a new range of $f_{f}^{F}$ is found, as is represented in Table 4.

Table 4 shows how the $f_{f}^{F}$ increments with the increase in the percentage of MAPE, as was expected. The addition of the coupling agent enhances the strength of the interface between the matrix and the natural fiber; therefore, the factor $f_{f}^{F}\;$ must follow the same pattern. It arrives at the maximum value at the same percentage 8 wt% of MAPE as it does the flexural strength of the composite. The obtained results used an estimation of β = 0.15 for the flexural strength. When using a different value of β, for example, β = 0.175, the result delivered by the model is a mean value of $\sigma_{f}^{F}$ = 746 ± 71.1 MPa, a value that is more approximate to the results obtained with the other two models, 775 MPa and 785.1 MPa. However, the value of β = 0.175 deviates from the original value found in the literature of β = 0.1 [50]. All the knowledge about the value of β alludes to tensile cases. With no information about the efficiency factor of strengths between fiber and matrix for flexural strength cases in previous works, there is a need for further research on this matter.

#### 4.4.2. Modeling of the Composite regarding AF Content

By modeling the composite with different contents of MAPE we verified the reliability of the results obtained with the three methods described before. Afterward, we proceeded to perform the same procedure for the rest of the samples with different AF percentages using the same equations to evaluate the samples as with the intrinsic flexural strength of the composite BioPE + 30% AF. We used the methodology described before to solve the mRom. To determine the value of $\sigma_{f}^{F}$, we used the three models with their respective Equations (4), (6), and (7). Table 5 shows the obtained results.

The mean value of $\sigma_{f}^{F}$ is obtained using the Hashemi approach, giving a result of 744.6 MPa. We used every value of $\sigma_{t}^{c}$ and $\sigma_{f}^{c}$ for every sample in order to obtain $\sigma_{t}^{F}$ for each one. Afterward, we obtained a ratio value for every sample. The mean value of the ratio was 1.45. This ratio is consistent with the ratio described in Hashemi’s publication, which was 1.5 [61]. Using Equation (6) with the ratio 1.45 and the mean value of $\sigma_{t}^{F}$ = 515.35, we obtained $\sigma_{f}^{F}$ = 747.25 MPa. This outcome is in line with the result of the mean value calculated for every single AF content, 744.6 MPa.

We used the hypothesis in which we ruled out the contribution of the matrix and assumed that the tensile–flexural strength ratio of the composite is the same as the tensile–flexural strength ratio of the fiber. The equation for the contribution of the fiber gives the result of a mean value $\sigma_{f}^{F}$ = 786.8 MPa, where the values of $\sigma_{t}^{F}$ by different AF percentages were obtained in a previous publication [52].

With the Hirsch equation, the mean value of the $\sigma_{f}^{F}\;$ is 863.5 ± 82.6 MPa. Various authors use the Hirsch model to determine the flexural properties by modifying the rule of mixtures for Young’s modulus [53,66], but none of the publications about flexural strength or flexural modulus determine the value of *β* or the efficiency factor of strengths between fiber and matrix, and nor deliver any indication about how to establish them. The main issue with using the Hirsch equation is evaluating this factor β. To verify the reliability of the results, the Hirsch equation (Equation (4)) was used for modeling the mean values of flexural and tensile strength for all the samples.

**Figure 11:**
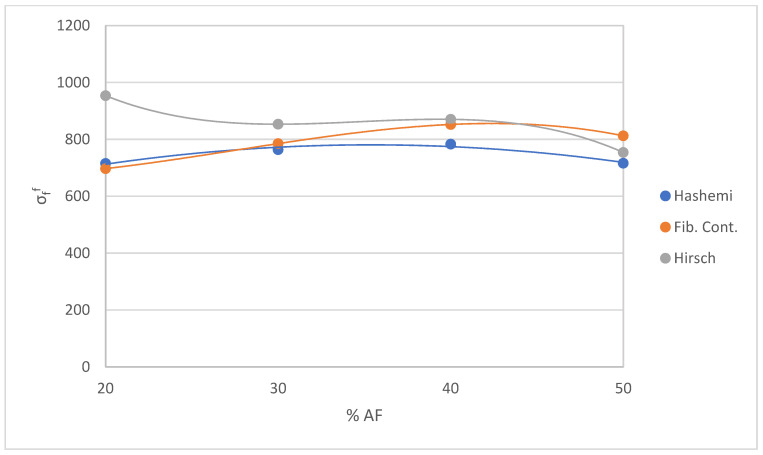
Effect of AF content on the intrinsic fiber strength, according to the three methods of modeling.

An estimation of β = 0.15 was used in the calculations of the flexural strength. All three results obtained are similar (744.6 MPa, 786.8 MPa, and 863.5 ± 82.6 MPa), meaning the estimation made was correct. The standard deviation of the value obtained through the Hirsch approach must be taken into consideration to verify that the results intersect. In the Hirsch case, the similarity is obtained because of the low value of the deviation. Figure 11 shows the impact of AF content on the theoretical values of the intrinsic strength of the fibers.

The value of $f_{f}^{F}$ has been determined for the three methods using Equation (2) of the mRom. The mean value of $f_{f}^{F}$ is 0.22 according to the Hashemi approach, the mean value of $f_{f}^{F}$ by the fiber contribution approach is 0.21, and, in compliance with the Hirsch approach, the mean value of $f_{f}^{F}$ is 0.19. Their values range from 0.19 to 0.22, as was expected. After all these verifications, we deem it necessary to conduct further research to set a value of β and, furthermore, to determine if the same value of factor β can be applied for the tensile and the flexural cases.

## 5. Conclusions

This investigation focused on the flexural strength and deformation of abaca fiber and BioPE composite. The results revealed that the composites comprising abaca fiber with a BioPE matrix exhibited superior flexural properties compared to BioPE and HDPE alone. Even without the addition of a coupling agent, the flexural strength increased by approximately 35% compared to HDPE and 40% compared to BioPE.

Furthermore, it was established that the addition of MAPE further enhanced the flexural strength of the composite. The addition of 8 wt% MAPE improved the properties by enhancing the interface between the reinforcement and the matrix. The flexural strength of the composite was two and a half times higher than that of BioPE and HDPE alone and more than one and a half times higher than the composite without any MAPE content.

Increasing the amount of abaca fiber in the composite led to improved flexural properties. However, beyond a 50 wt% content of AF, the properties began to decrease, indicating that this amount represented the optimal level. The increased presence of abaca bundles at higher fiber content hindered proper matrix infiltration and resulted in the appearance of voids at the interface. Additionally, a significant decrease in the material’s elongation at maximum force of 68% was observed.

To determine the intrinsic flexural strength of the fiber, a three-method model based on the approaches of Hashemi, fiber contribution, and Hirsch was developed. This model demonstrated reliability and yielded consistent results. However, further research is needed to investigate the value of *β* in flexural cases.

Further research will be centered on the flexural modulus and its evolution according to fiber content and MAPE percentage.

## Figures and Tables

**Table 1:** Flexural strength of BioPE composites reinforced with a 30% content of abaca.

Samples	$V^{F}$	$\sigma_{f}^{C}$ (MPa)	D (mm)	$\varepsilon_{f}^{c}$ (%)	$\sigma_{f}^{m*}$ (MPa)
HDPE	0	22.28 ± 0.39	10.51 ± 0.17	7.1	22.28
BioPE	0	21.25 ± 0.27 ^a^	9.8 ± 0.04 ^e^	6.6	21.25
BioPE + 30% Abaca + 0% MAPE	0.218	30.11 ± 0.71 ^b^	5.04 ± 0.10 ^a^	3.4	16.73
BioPE + 30% Abaca + 2% MAPE	0.218	39.01 ± 0.75 ^c^	5.19 ± 0.04 ^a^	3.5	16.95
BioPE + 30% Abaca + 4% MAPE	0.218	46.55 ± 0.18 ^d^	5.48 ± 0.02 ^b^	3.7	17.37
BioPE + 30% Abaca + 6% MAPE	0.218	48.31 ± 0.77 ^e^	5.94 ± 0.05 ^c^	4.0	18.00
BioPE + 30% Abaca + 8% MAPE	0.218	50.28 ± 0.63 ^f^	6.34 ± 0.11 ^d^	4.3	18.53
BioPE + 30% Abaca + 10% MAPE	0.218	48.56 ± 0.41 ^e^	5.99 ± 0.10 ^c^	4.0	18.00

Different letters, ^a, b, c, d, e^, and ^f^, represent the statistical differences (ANOVA, *p* < 0.05) between the properties of the materials.

**Table 2:** Flexural properties of BioPE composites reinforced with abaca, with an 8% content of MAPE.

Samples	$V^{F}$	$\sigma_{f}^{C}$ (MPa)	D (mm)	$\sigma_{f}^{m*}$ (MPa)	$\varepsilon_{f}^{c}$ (%)
HDPE	0	22.28 ± 0.39	10.51 ± 0.17	22.82	7.1
BioPE	0	21.25 ± 0.33	9.8 ± 0.05	21.25	6.6
BioPE + 20% Abaca + 8% MAPE	0.140	41.74 ± 0.64	7.45 ± 0.08	19.38	5.0
BioPE + 30% Abaca + 8% MAPE	0.218	50.28 ± 0.45	6.34 ± 0.10	18.53	4.3
BioPE + 40% Abaca + 8% MAPE	0.302	59.62 ± 0.32	4.12 ± 0.23	15.14	2.8
BioPE + 50% Abaca + 8% MAPE	0.394	64.23 ± 0.81	3.12 ± 0.06	13.13	2.1

**Table 3:** The flexural intrinsic strength of fibers of the BioPE composites reinforced with a 30% content of abaca, according to the three models.

	Hashemi		Contrib. of Fiber		Hirsch	
Samples	$\sigma_{f}^{F}$ (MPa)	$f_{f}^{F}$	$\sigma_{f}^{F}\;$ (MPa)	$f_{f}^{F}$	$\sigma_{f}^{F}\;$ (MPa)	$f_{f}^{F}$
BioPE + 30% Abaca + 0% MAPE	689.4	0.113	785.1	0.100	312.8	0.25
BioPE + 30% Abaca + 2% MAPE	803.4	0.147	785.1	0.150	573.3	0.206
BioPE + 30% Abaca + 4% MAPE	838.2	0.180	785.1	0.193	787.3	0.192
BioPE + 30% Abaca + 6% MAPE	788.2	0.199	785.1	0.200	818.1	0.192
BioPE + 30% Abaca + 8% MAPE	763.9	0.215	785.1	0.209	858.9	0.191
BioPE + 30% Abaca + 10% MAPE	767.0	0.206	785.1	0.202	825.7	0.192

**Table 4:** The flexural intrinsic strength of fibers of the BioPE composites reinforced with a 30% content of abaca and using the optimal $\sigma_{f}^{F}$, according to the Hirsch model.

	Hirsch	
Samples	$\sigma_{f}^{F}\;$ (MPa)	$f_{f}^{F}$
BioPE + 30% Abaca + 0% MAPE	858.9	0.091
BioPE + 30% Abaca + 2% MAPE	858.9	0.138
BioPE + 30% Abaca + 4% MAPE	858.9	0.176
BioPE + 30% Abaca + 6% MAPE	858.9	0.183
BioPE + 30% Abaca + 8% MAPE	858.9	0.191
BioPE + 30% Abaca + 10% MAPE	858.9	0.184

**Table 5:** The flexural intrinsic strength of fibers of the BioPE composites reinforced with an 8 wt% percentage of MAPE, according to the three models.

	**Hashemi**		Contrib. of Fiber		Hirsch	
**Samples**	$\sigma_{f}^{F}\;$ (MPa)	$f_{f}^{F}$	$\sigma_{f}^{F}\;$ (MPa)	$f_{f}^{F}$	$\sigma_{f}^{F}\;$ (MPa)	$f_{f}^{F}$
BioPE + 20% Abaca + 8% MAPE	715.5	0.250	697.2	0.257	959.4	0.187
BioPE + 30% Abaca + 8% MAPE	763.9	0.215	785.1	0.209	858.9	0.191
BioPE + 40% Abaca + 8% MAPE	783.0	0.207	852.4	0.191	877.1	0.185
BioPE + 50% Abaca + 8% MAPE	716.2	0.199	812.5	0.176	758.5	0.188

## Data Availability

Not applicable.
